# Competition between *Burkholderia pseudomallei* and *B. thailandensis*

**DOI:** 10.1186/s12866-015-0395-7

**Published:** 2015-03-03

**Authors:** Wikanda Ngamdee, Sarunporn Tandhavanant, Chanthiwa Wikraiphat, Onrapak Reamtong, Vanaporn Wuthiekanun, Jeanne Salje, David A Low, Sharon J Peacock, Narisara Chantratita

**Affiliations:** Department of Microbiology and Immunology, Faculty of Tropical Medicine, Mahidol University, 420/6 Rajvithi Road, Bangkok, 10400 Thailand; Mahidol-Oxford Tropical Medicine Research Unit, Faculty of Tropical Medicine, Mahidol University, Bangkok, Thailand; Department of Molecular Tropical Medicine and Genetics, Faculty of Tropical Medicine, Mahidol University, Bangkok, Thailand; Department of Molecular, Cellular, and Developmental Biology, University of California, Santa Barbara, CA USA; Biomolecular Science and Engineering Program, University of California, Santa Barbara, CA USA; Department of Medicine, University of Cambridge, Addenbrooke’s Hospital, Cambridge, UK

**Keywords:** *Burkholderia pseudomallei*, *B. thailandensis*, Melioidosis, Swarming, Flagella, Competitive growth inhibition

## Abstract

**Background:**

*Burkholderia pseudomallei* is a Gram-negative bacterium that causes melioidosis, an often fatal disease in tropical countries. *Burkholderia thailandensis* is a non-virulent but closely related species. Both species are soil saprophytes but are almost never isolated together.

**Results:**

We identified two mechanisms by which *B. pseudomallei* affects the growth of *B. thailandensis.* First, we found that six different isolates of *B. pseudomallei* inhibited the growth of *B. thailandensis* on LB agar plates. Second, our results indicated that 55% of isolated strains of *B. pseudomallei* produced a secreted compound that inhibited the motility but not the viability of *B. thailandensis*. Analysis showed that the active compound was a pH-sensitive and heat-labile compound, likely a protein, which may affect flagella processing or facilitate their degradation. Analysis of bacterial sequence types (STs) demonstrated an association between this and motility inhibition. The active compound was produced from *B. pseudomallei* during the stationary growth phase.

**Conclusion:**

Taken together, our results indicate that *B. pseudomallei* inhibits both the growth and motility of its close relative *B. thailandensis*. The latter phenomenon appears to occur via a previously unreported mechanism involving flagellar processing or degradation.

**Electronic supplementary material:**

The online version of this article (doi:10.1186/s12866-015-0395-7) contains supplementary material, which is available to authorized users.

## Background

*B. pseudomallei* is a Gram-negative bacillus and the cause of melioidosis. Most cases of infection are reported from northeast Thailand and northern Australia, although an increasing number of cases are being reported from across Southeast Asia, and from Africa and south America [1]. *B. pseudomallei* is an environmental saprophyte, and infection arises following bacterial inoculation, inhalation or ingestion [2]. The range of clinical manifestations are broad, but the majority of patients present with an acute febrile illness associated with one or more of pneumonia, bacteremia, or abscess formation in the spleen, liver or elsewhere [3]. In Thailand this infection accounts for 20% of community-acquired septicemias [3], and the mortality rate is 12-40% despite appropriate antimicrobial therapy.

Environmental surveys to detect *B. pseudomallei* have been performed to address a range of questions, including mapping of geographic distribution, the effect of sampling depth and season on positivity of *B. pseudomallei* in soil and water, and the phylogeny of environmental isolates [4]. A striking observation arising from an intensive sampling survey conducted in a single plot of disused land (237.5 m^2^) in northeast Thailand was the genetic diversity of *B. pseudomallei* within and between sampling points. Genotyping of 600 primary culture plate colonies from 3 sampling points showed that each contained three or four different *B. pseudomallei* clones, with little overlap in genotype between samples but a predominance of a single genotype within a given sample [4]. One explanation for bacterial population structuring within a single sample is that the predominant clone has a competitive advantage over other *B. pseudomallei* lineages and/or other microbial species or genera.

*B. pseudomallei* is genetically closely related to the non-pathogenic *B. thailandensis* [5,6], which is also present in the environment including geographic areas that are positive for *B. pseudomallei*. Although *B. thailandensis* is rarely searched for systematically during environmental surveys of *B. pseudomallei*, *B. thailandensis* grows on the same culture media and the colony morphology on agar can be difficult to distinguish from *B. pseudomallei*. As a result, *B. thailandensis* is frequently isolated during soil sampling and discarded after identification. A review of environmental study indicates that *B. pseudomallei* and *B. thailandensis* are rarely isolated from the same sampling location [7]. A study 232 soil isolates from 4 regions of Thailand reported that the ratio of *B. pseudomallei* to *B. thailandensis* was 1.7, 0.9, 0.5 and 0.4 in northeastern, southern, northern and central regions, respectively [7]. The higher ratio of *B. pseudomallei* and *B. thailandensis* in the northeast was associated with a higher prevalence of melioidosis in this region compared with others. One explanation for this observation is competition between *B. pseudomallei* and *B. thailandensis.*

Several studies have provided evidence for a competitive advantage of *B. thailandensis* over other environmental bacterial species through a range of mechanisms. *B. thailandensis* secretes an antibiotic that inhibits the growth of *Bacillus subtilis* [8]. Inactivation of type VI secretion systems (T6SS)-1 renders *B. thailandensis* more susceptible to cell contact-induced stasis by *Pseudomonas putida*, *Pseudomonas fluorescens* and *Serratia proteamaculans. B. thailandensis* lacking T6SS-1 is also rapidly displaced from mixed biofilms with *P. putida*, whereas wild-type persists and overgrows the competitor [9]. *B. pseudomallei* and *B. thailandensis* have also been reported to have a contact-dependent growth inhibition (CDI) system mediated by the CdiB/CdiA family of two-partner secretion proteins [10,11].

The aim of this study was to use in vitro assays to investigate competition (including viability and motility) between a range of *B. pseudomallei* and *B. thailandensis* isolates associated with human infection (*B. pseudomallei*) and the environment (both species), and to explore the relationship between inhibition and genotype.

## Results

### Growth rate analysis of *B. pseudomallei* and *B. thailandensis*

Different growth rates may affect the number of viable bacteria in the growth inhibition assay. Thus, prior to observation of competition between *B. pseudomallei* and *B. thailandensis*, the individual growth of six *B. pseudomallei* (H1244a, 1106b, 1026b, B4, 1710a and K96243) and *B. thailandensis* Bt6 were compared in LB broth. Using a starting inoculum of 1 × 10^5^ cfu/ml, log and stationary phase occurred at 2 h and 12 h, respectively, for all isolates. There was no difference in doubling time between *B. pseudomallei* and *B. thailandensis*. The average doubling time for *B. pseudomallei* K96243, H1244a, 1106b, 1026b, 1710a and B4 were 38.7, 38.3, 38.4, 38.4, 38.6 and 38.5, and for *B. thailandensis* Bt6 was 39.8 min, respectively.

### *B. pseudomallei* inhibits growth of *B. thailandensis*

**Figure 1:**
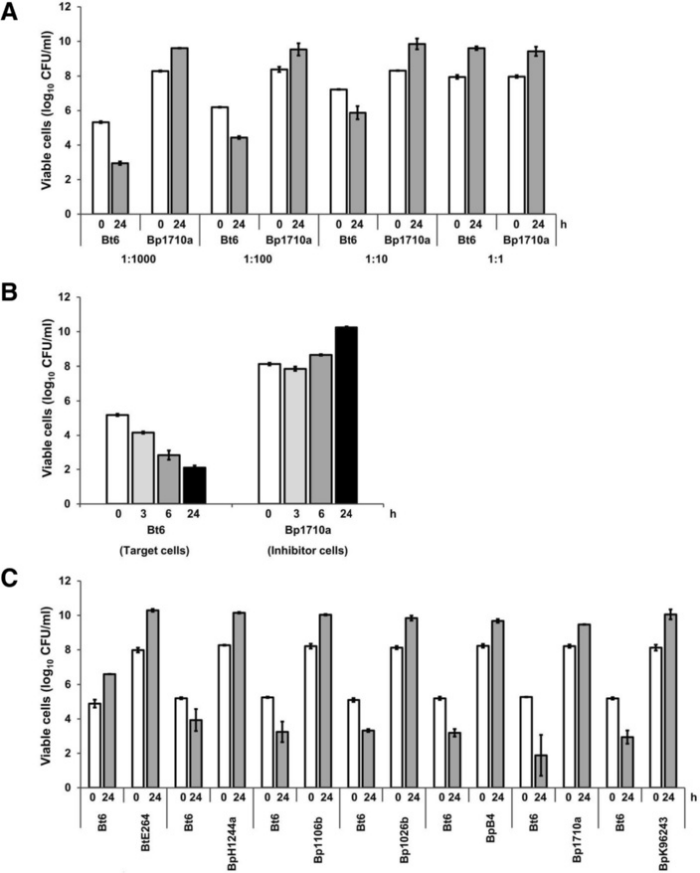
**Growth inhibition of** ***B. thailandensis*** **Bt6 by** ***B. pseudomallei*** **1710a on LB agar at 37°C. (A)** *B. thailandensis* Bt6 (target) and *B. pseudomallei* 1710a (inhibitor) cells were mixed at different inhibitor to target cell ratios and viable counts were measured at 0 h and 24 h. **(B)** Time Course. Viable cell counts were determined at the indicated times using an inhibitor to target ratio of 1000:1 **(C)** Growth competition analysis of *B. thailandensis* Bt6 with six *B. pseudomallei* isolates was carried out at inhibitor to target cell ratios of 1000:1 at 0 h and 24 h. A control growth competition was carried out between *B. thailandensis* Bt6 and *B. thailandensis* E264.

Using the growth inhibition assay, we found that *B. pseudomallei* 1710a inhibited *B. thailandensis* Bt6 when mixed at ratios from 1000:1 to 10:1 (Figure 1A). At 24 h of incubation, the number of viable *B. pseudomallei* increased by 1–1.5 logs and the number of *B. thailandensis* decreased by 1.5-2.0 logs. There was no inhibition at a ratio of 1:1. The reduction in number of *B. thailandensis* Bt6 after co-culture with *B. pseudomallei* 1710a was maximal at a ratio of 1000:1.

Next, we examined inhibition at different incubation time points using a fixed ratio of *B. pseudomallei* 1710a and *B. thailandensis* Bt6 of 1000:1. The number of viable bacteria for *B. pseudomallei* and *B. thailandensis* at 3 h, 6 h and 24 h following co-culture was inversely related, with levels of *B. pseudomallei* increasing and *B. thailandensis* decreasing over time (Figure 1B). Maximum inhibition was observed at 24 h of incubation. There was no increased inhibition or increased growth of Bt6 after 48 h (data not shown).

We examined whether other *B. pseudomallei* isolates could inhibit growth of *B. thailandensis* Bt6. The co-culture was repeated for each of five clinical *B. pseudomallei* isolates (K96243, H1244a, 1106b, 1026b, 1710a) and one soil *B. pseudomallei* isolate (B4) against *B. thailandensis* Bt6 at a ratio of 1000:1. This demonstrated a 1 to 2 log increase for six different *B. pseudomallei* isolates and a 1 to 3 log decrease in *B. thailandensis* Bt6 (Figure 1C). The highest level of inhibition was observed with the pair *B. pseudomallei* 1710a and *B. thailandensis* Bt6. These data suggest that multiple *B. pseudomallei* isolates are able to inhibit the growth of *B. thailandensis*.

### *B. pseudomallei* inhibits *B. thailandensis* swarming motility via a growth-independent inhibition mechanism

**Figure 2:**
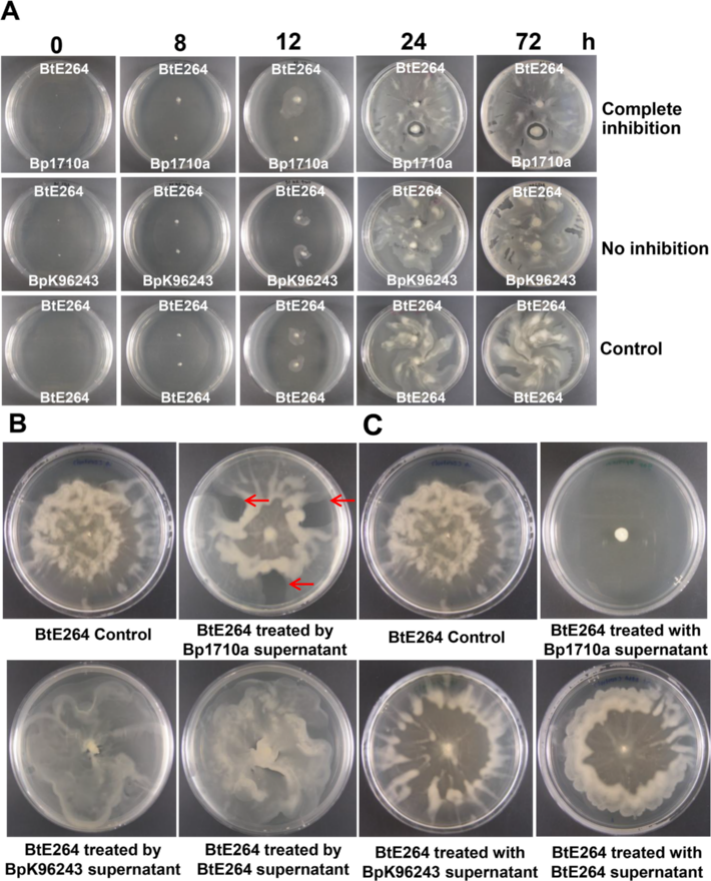
**Interaction between** ***B. pseudomallei*** **and** ***B. thailandensis*** **pairs on swarm agar.** Three methods were used to detect interactions between the two species. **(A)** *B. pseudomallei* and *B. thailandensis* were spotted on different poles of a swarm agar plate and the growth observed daily over a 72 h incubation time course. An example of “Complete inhibition” is shown in which a clear zone was present around the Bp1710a strain. In contrast, no clear zone was observed around BpK96243 (“No inhibition”) or control strain *B. thailandensis* E264. **(B)** Inhibitory effect of *B. pseudomallei* cell-free supernatant on *B. thailandensis* E264 motility on swarm agar. A *B. thailandensis* colony was spotted onto the center of swarm agar and incubated at 37°C for 16 h to allow initial swarming to begin, after which three drops of *B. pseudomallei* cell-free supernatant were inoculated at 2, 6 and 10 o’clock followed by further incubation for 16 hours. Swarming of *B. thailandensis* E264 was blocked around three spots of supernatant from *B. pseudomallei* 1710a supernatant (arrows), and is representative of results using strains that inhibited *B. thailandensis* swarming. *B. pseudomallei* K96243 is representative of strains that did not inhibit *B. thailandensis* swarming. Controls: *B. thailandensis* E264 colony with no supernatant added (top left panel), and *B. thailandensis* E264 colony with *B. thailandensis* E264 supernatant added (bottom right panel). **(C)** The inhibitory effect of *B. pseudomallei* cell-free supernatant on *B. thailandensis* E264 colony swarming. One hundred microliters of cell-free supernatant from *B. pseudomallei* was deposited at the center of a swarm agar plate and left to dry in air for 15 min. Subsequently, *B. thailandensis* were spotted at the center of the plate. Growth of the swarm colony was observed after incubation at 37°C for 18 h.

The second part of our study was initiated by the observation that in the growth inhibition assay described above, a colony of *B. thailandensis* Bt6 on LB agar failed to grow to the edge of an adjacent colony of *B. pseudomallei* 1710a following co-culture. Using a swarm plate assay, sixty-seven *B. pseudomallei* isolates were tested against each of five *B. thailandensis* isolates, in which *B. pseudomallei* and *B. thailandensis* were spotted on different poles of an agar plate (Methods, method (i)). We observed that *B. thailandensis* swarmed more rapidly than *B. pseudomallei,* and classified the interaction between the two species into two types as follows: (1) evidence of inhibition, with a complete or partial zone of inhibition around the *B. pseudomallei* colony and mounding of *B. thailandensis* growth surrounding this zone (e.g. strain 1710a, Figure 2A); or (2) no evidence of inhibition, with *B. thailandensis* swarming over the entire *B. pseudomallei* colony (e.g. strain K96243, Figure 2A). Thirty-seven out of sixty-seven *B. pseudomallei* showed evidence of inhibitory activity against *B. thailandensis*. A consistent result was observed for a given *B. pseudomallei* isolate against each of the five *B. thailandensis* isolates.

We next explored whether the inhibition of colony migration observed was due to a secreted factor (method ii). We tested the cell-free supernatants of sixty-seven *B. pseudomallei* isolates for the inhibition of swarming of five *B. thailandensis* isolates. Inhibition was evident for the same thirty-seven *B. pseudomallei* isolates (55.2%) defined by method (i), and as before each *B. pseudomallei* isolate gave a consistent inhibition result to each of the five *B. thailandensis* isolates (Additional file 1: Table S1). When inhibition occurred, the zone containing cell-free *B. pseudomallei* supernatant was clear and was surrounded by piled up *B. thailandensis* (Figure 2B).

A further experiment was performed to explore these findings using the same sixty-seven *B. pseudomallei* isolates. When cell-free supernatant of *B. pseudomallei* was spotted onto swarm agar, left to dry for 15 min and then overlaid by *B. thailandensis* cells (method iii), we observed two outcomes; *B. thailandensis* failed to swarm when overlaid on supernatant from the same thirty-seven *B. pseudomallei*, while in the remainder *B. thailandensis* swarmed without evidence of inhibition (Figure 2C). Again, each *B. pseudomallei* showed the same results for five different *B. thailandensis* isolates. Based on these results, we divided *B. pseudomallei* into two groups based on the presence or absence of swarm inhibitory activity.

### Cell-free supernatant of *B. pseudomallei* does not inhibit growth of *B. thailandensis*

Since the supernatant of *B. pseudomallei* 1710a inhibited *B. thailandensis* E264 swarming motility, we sought to investigate whether the supernatant of *B. pseudomallei* affected viability of *B. thailandensis*. A broth microdilution assay was used to measure growth inhibition. We found that the number of viable *B. thailandensis* cells did not decrease during incubation at 37°C during exposure to different concentrations of cell-free supernatant from *B. pseudomallei* 1710a, *B. pseudomallei* K96243 and *B. thailandensis* E264 control, and the number of treated *B. thailandensis* E264 cells counts were comparable to that of the untreated control (data not shown). This result suggests that the secreted factor (denoted here as “inhibitory factor”) does not function as growth inhibitor but only inhibited *B. thailandensis* swarming.

### Inhibitory activity is associated with genotype

The *B. pseudomallei* multilocus sequence type (ST) was known for sixty-six out of sixty-seven isolates. The frequency of inhibition was defined for each ST, which demonstrated an association between the two groups. Inhibition was observed in 14 STs represented by 36 isolates, but not in 10 STs represented by 30 isolates. However, 5 STs contained both inhibitory and non-inhibitory isolates (Table 1). This included 12/13 of ST54 that were able to inhibit *B. thailandensis*, and 18/20 of ST70 lacking swarm inhibitory activity (P <0.001).

**Table 1:** **Sequence type (ST) of 67** ***B. pseudomallei*** **isolates and their corresponding inhibitory effect on** ***B. thailandensis*** **motility**

**ST**	**Total tested**	Inhibition present* (n=37)	Inhibition absent* (n=30)
10	1	0	1
15	1	1	0
33	1	1	0
54	13	12	1
60	9	6	3
70	20	2	18
93	1	1	0
102	1	1	0
126	2	0	2
129	1	0	1
132	1	1	0
163	1	1	0
176	2	1	1
177	7	6	1
185	1	1	0
211	1	1	0
304	1	0	1
424	1	0	1
501	1	1	0
Unknown (strain 164)	1	1	0
**Total ST**		**14 STs**	**10 STs**

*Inhibition effect of *B. pseudomallei* cell-free supernatant on five *B. thailandensis* isolates which showed the same result for each *B. pseudomallei* isolate. The inhibition of *B. thailandensis* swarming was performed by all three types of assays which gave the same results.

### Inhibitory *B. pseudomallei* strains were isolated from different sources

By comparing the effect on *B. thailandensis* motility on swarm agar, we did not find any significant difference between clinical and environmental isolates. Inhibition was found in 20 of 39 clinical (51.3%) and 17 of 28 environmental isolates (60.7%) (p=0.44). The geographical origin of *B. pseudomallei* demonstrating inhibition included Thailand (36/63, 57.1%) and Australia (1/4, 25.0%) (p=0.32).

### Swarm inhibitory activity is found in dominant and minor populations of *B. pseudomallei* in the same soil samples

We previously demonstrated genetic diversity of *B. pseudomallei* in a single soil sample, with a predominant genotype co-existing with two or three other genotypes [4]. We hypothesized that this structuring may be influenced by the ability of some strains to secrete an inhibitory factor. We examined the inhibition of *B. thailandensis* motility by cell-free *B. pseudomallei* supernatant for each of the STs identified from 3 independent soil samples [4] (Table 2). Only two isolates failed to demonstrated swarm inhibition, one from each of two soil samples. This evidence does not support our hypothesis, although there may be other effects that would not be detected by this assay.

**Table 2:** **Inhibition of** ***B. thailandensis*** **motility by cell-free supernatant of** ***B. pseudomallei*** **from three independent soil samples**

**Soil sample** ^**a**^	**Bp isolates**	**Sequence type**	**No. of colonies** ^**b**^	**Inhibition** ^**c**^
E4	A1	ST424	38 (19%)	-
	A2	ST177	12 (6%)	+
	A3	ST176	10 (5%)	+
	A4	ST185	140 (70%)	+
D10	B1	ST33	50 (25%)	+
	B2	ST60	18 (9%)	+
	B3	ST163	103 (51.5%)	+
	B4	ST176	29 (14.5%)	+
A11	C1	ST93	174 (87%)	+
	C2	ST304	17 (8.5%)	-
	C4	ST60	9 (4.5%)	+

^a^, soil sample and culture data was obtained from our previous study [4]. The study reported the distribution of *B. pseudomallei* within an area of disused land in northeast Thailand and genotypes of primary plate colonies isolated from three independent sampling points (E4, D10 and A11).

^b^, Out of a total of 200 colonies per sample.

^c^, Inhibition effect of *B. pseudomallei* cell-free supernatant on five *B. thailandensis* isolates. The inhibition of *B. thailandensis* swarming was performed by the method (ii) assay. The table shows results of five *B. thailandensis* isolates which had the same results.

### No inhibition between pairwise testing of different *B. pseudomallei* isolates

We further investigated the possibility that a *B. pseudomallei* predominant genotype in a single soil sample may use the inhibition activity to limit the other minor genotypes of the same species. Pairwise testing was examined of the inhibition of *B. pseudomallei* motility by cell-free *B. pseudomallei* supernatant for each of the STs identified from 3 independent soil samples [4]. The *B. pseudomallei* isolates analyzed are shown in Table 2. We found that the culture supernatant of all *B. pseudomallei* isolated from soil did not inhibit motility of any other *B. pseudomallei*. We also tested the supernatant of a clinical isolate (*B. pseudomallei* 1710a) against itself and 66 *B. pseudomallei* other isolates (listed in Additional file 1: Table S1) and no inhibition were observed. This suggests that the role of this secreted factor may be to inhibit the motility of other species.

### Three isogenic morphotypes showed the same inhibition activity results

We previously reported that *B. pseudomallei* can switch colony morphology to different types under stress conditions [12,13]. We next investigated whether the secretion of inhibitory factor in a given isolate is related to colony morphotype. Three isogenic morphotypes referred as types I, II and III generated from wild-type type I for each five *B. pseudomallei* strains (153, 164, K96243, B3 and B4) were tested against each of five *B. thailandensis* strains (E29, E175, E264, E421 and E426) using the inhibition assay method (ii). *B. pseudomallei* strains 153, 164 and K96243 were clinical isolates and B3 and B4 were soil isolates. All three colony morphotypes of strains 153, 164 and B4 showed inhibition, whilst all three types of K96243 and B3 did not. This result indicated that the secretion of inhibitory factor by *B. pseudomallei* is strain-dependent but not related to the morphotypes tested.

### *B. pseudomallei* secretes an inhibitory factor during stationary phase

**Figure 3:**
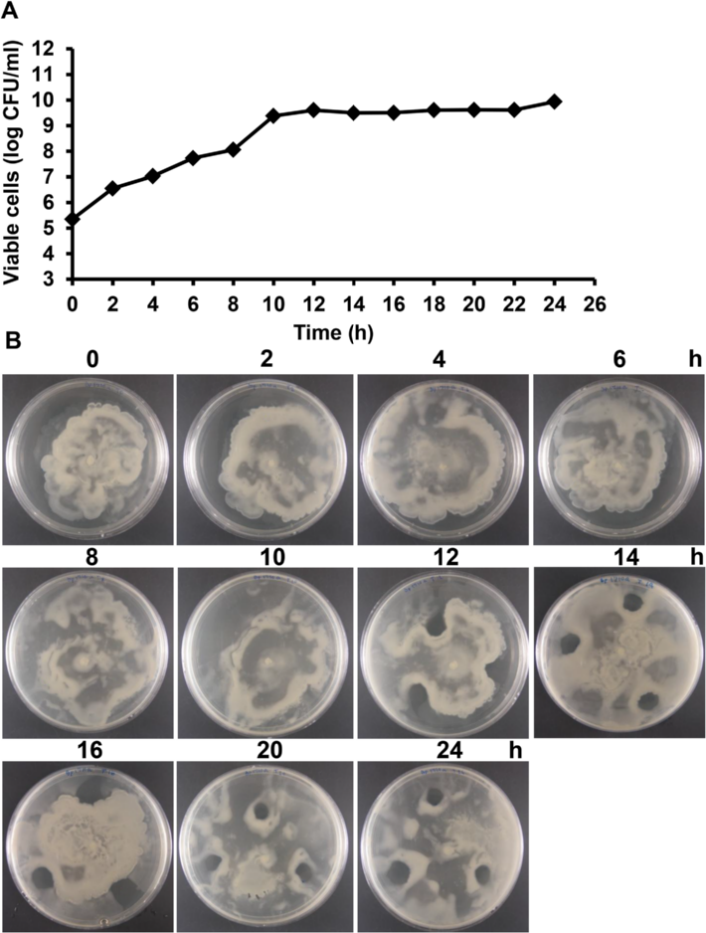
**Inhibitory activity of cell-free supernatant of** ***B. pseudomallei*** **1710a produced during growth in LB broth. (A)** Growth curve of *B. pseudomallei* 1710a, **(B)** Inhibition activity of cell-free supernatant from *B. pseudomallei* 1710a which was collected at 2 h time interval during culture, with *B. thailandensis* E264 motility on swarm agar.

To determine whether the secretion of the *B. pseudomallei* inhibitory factor was growth-phase dependent, we cultured 1 × 10^5^ cfu/ml *B. pseudomallei* 1710a in 10 ml LB broth at 37°C with shaking at 200 rpm for 24 h. The supernatant was collected every 2 h and filter-sterilized, and these samples examined for the presence of inhibition of *B. thailandensis* E264 swarming using inhibition assay method (ii). Inhibitory activity was detected in all aliquots collected after 12 h incubation when the *B. pseudomallei* count reached 5 × 10^9^ cfu/ml and was in stationary phase (Figure 3). This suggests that inhibitory factor secreted by *B. pseudomallei* may be concentration dependent or bacterial density-dependent.

### Estimation of the mass of the inhibitory factor of *B. pseudomallei*

**Figure 4:**
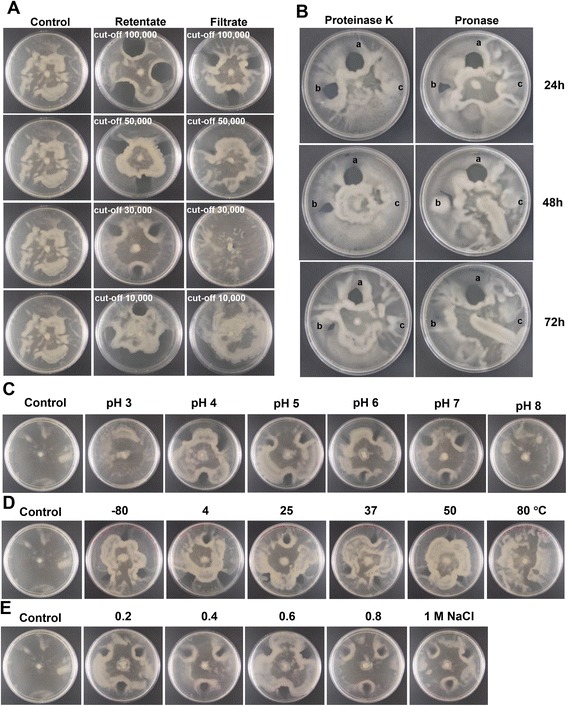
**Inhibitory activities on** ***B. thailandensis*** **E264 motility on swarm agar of cell-free supernatant of** ***B. pseudomallei*** **1710a after filtration, protein digestion, or incubation under different conditions. (A)** Inhibition zone in retentate and filtrate fractions following filtration through membranes with different molecular weight cut-off sizes. **(B)** Inhibitory activity of cell-free supernatant of *B. pseudomallei* 1710a after treatments with proteinase K (left panel) and pronase (right panel), **(a)** no treatment control, **(b)** treatment with proteinase K or pronase, **(c)** Enzymes alone (no supernatants) were spotted onto the agar in the same concentration as a control. **(C)** Inhibition activity of cell-free supernatant of *B. pseudomallei* 1710a following incubation at different pH at 37°C for 24 h. **(D)** Inhibition activity of cell-free supernatant of *B. pseudomallei* 1710a following incubation at different temperatures for 24 h. **(E)** Inhibition activity of cell-free supernatant of *B. pseudomallei* 1710a following incubation at different salt concentrations at 37°C for 24 h. The controls for **(A)**, **(C)**, **(D)** and **(E)** are *B. thailandensis* E264 swarming without the inoculation of cell-free supernatant. Plates were incubated at 37°C for 24 h.

Filtration through membranes with different pore sizes (10 kDa to 100 kDa size cut-off) was used to estimate the molecular weight of the inhibitory factor from *B. pseudomallei.* Two *B. pseudomallei* isolates with inhibition activity (1710a and 1026b) were tested. Following filtration, 15 times concentrated volume of original supernatants were obtained and 100 μl filtrate and retentate fractions were tested for the inhibition of *B. thailandensis* swarming using assay method (ii). The inhibition of *B. thailandensis* E264 swarming was detected in retentate samples from all membranes. The inhibition was not detected in the filtrate of both *B. pseudomallei* supernatants when 10 kDa and 30 kDa membranes were used, but was present in filtrate fractions from 50 kDa and 100 kDa membranes (Figure 4A). This result suggests that the inhibitory factor has an estimated MW of 30 to 50 kDa.

### Effect of protein digestion on inhibitory activity of *B. pseudomallei*

Next, we determined whether the inhibitory activity was mediated by a protein or non-protein factor. We tested the 15x-concentrated cell-free supernatant of *B. pseudomallei* 1710a on *B. thailandensis* E264 swarming, comparing the activity before and after protein digestion with each of two protease enzymes. The results shown in Figure 4B demonstrated that inhibitory activity was sensitive to proteinase K and pronase digestion. SDS-PAGE analysis demonstrated that proteinase K and pronase completely digested 1 mg BSA control. Thus, the inhibitory factor appears to be a protein.

### Stability of the inhibitory factor of *B. pseudomallei* under different conditions

We examined the effect of varying pH, temperature and salt concentration. Inhibitory activity of culture supernatant of *B. pseudomallei* 1710a against swarming of *B. thailandensis* E264 was retained in samples exposed to pH 4, 5, 6 and 7 but disappeared at pH 3 and 8, suggesting that the factor was inactivated at extreme pH (Figure 4C). The effect of heat on inhibitory activity was tested by incubating the *B. pseudomallei* culture supernatant at pH 7.0 at different temperatures for 24 h followed by testing in the inhibitory plate swam assay using method (ii). Incubation of supernatant at–80°C, 4°C, 25°C, 37°C and 50°C had no effect while the activity was lost when the sample was incubated at 80°C. The data indicates that the inhibition activity is heat-labile (Figure 4D). Variable salt concentrations achieved by adding NaCl to 0.2 M, 0.4 M, 0.6 M, 0.8 M and 1 M to the *B. pseudomallei* 1710a supernatant at pH 7.0 did not affect the inhibitory activity (Figure 4E).

### Inhibition is associated with motility defects but not flagella expression by *B. thailandensis*

**Figure 5:**
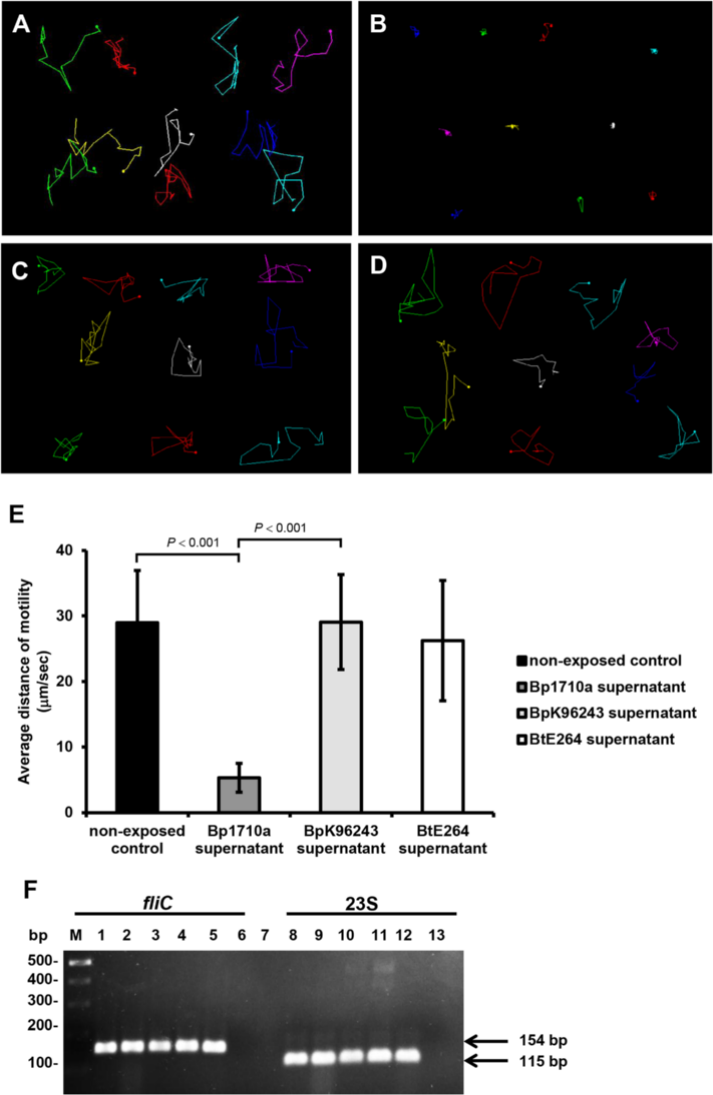
**Live-cell imaging analysis to track the movement of** ***B. thailandensis*** **E264. (A)** *B. thailandensis* E264 (BtE264); BtE264 exposed to cell-free supernatants from: **(B)**, *B. pseudomallei* 1710a; **(C)**, *B. pseudomallei* K96243 and **(D)**, *B. thailandensis* E264. Twenty cells were randomly tracked for movement by light microscopy and their paths of movement were determined from video recording using an ImageJ program (http://rsb.info.nih.gov/ij/). Colored lines show the paths of different cells over a 20 sec period. **(E)** Motility distance of *B. thailandensis* untreated BtE264 (non-exposed control) and after exposure to cell-free supernatants from *B. pseudomallei* 1710a, *B. pseudomallei* K96243 and *B. thailandensis* E264 control. **(F)** Expression of *fliC* RNA and 23S rRNA of *B. thailandensis* E264 exposed to cell-free supernatant of *B. pseudomallei*. RT-PCR of *fliC* RNA (lane 1–4) and 23S rRNA (lane 8–11) of *B. thailandensis* E264 colony exposed to cell-free supernatant of *B. pseudomallei* 1710a (lane 1 and 8), *B. pseudomallei* K96243 (lane 2 and 9), *B. thailandensis* E264 (lane 3 and 10), and non-exposed control (lane 4 and 11). Lanes 5 and 12 are PCR of genomic DNA controls for *fliC* and 23S rRNA primers respectively. Lanes 6 and 13 are no RT negative controls.

Under light microscopy and video capture, we observed that *B. thailandensis* E264 exposed to supernatant of *B. pseudomallei* 1710a (inhibitory strain) had reduced motility compared to that after exposure to supernatant from *B. pseudomallei* K96243 (non-inhibitory strain), *B. thailandensis* E264, or the unexposed control (Figure 5A-D). Measurement of motility showed a reduction in the average distance moved by *B. thailandensis* E264 cells exposed to supernatant of *B. pseudomallei* 1710a compared with the non-exposed control or exposure to supernatant from *B. pseudomallei* K96243 or *B. thailandensis* E264 (Figure 5E).

**Figure 6:**
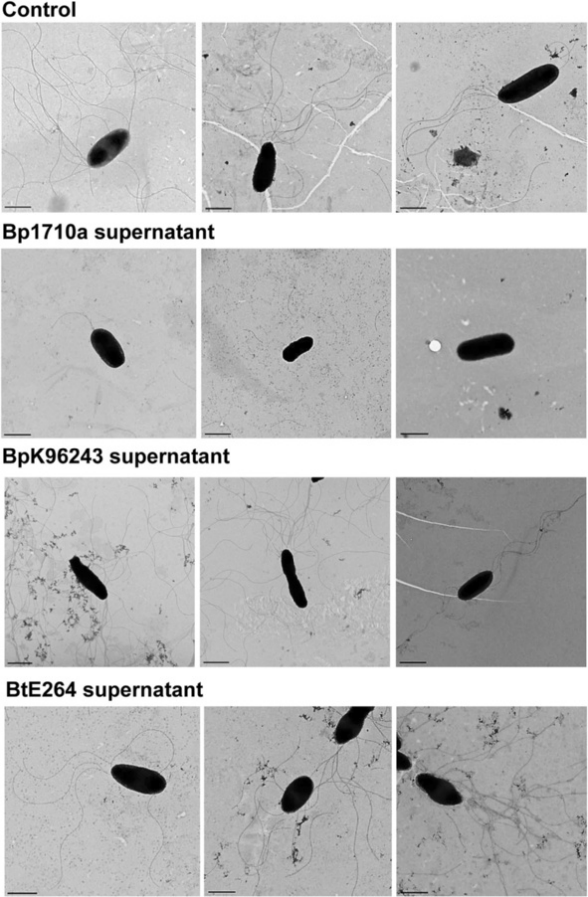
**Transmission electron microscopy (TEM) of** ***B. thailandensis*** **E264 unexposed control, or exposed to cell-free supernatant of** ***B. pseudomallei*** **1710a,** ***B. pseudomallei*** **K96243 or** ***B. thailandensis*** **E264, respectively.** Bacterial cells were negatively stained with 1% uranyl acetate and visualized by TEM. Scale bars illustrate 1 μm.

The effect on motility was further examined used transmission electron microscopy (TEM) to determine the proportion of flagellated bacteria. Following exposure to cell-free supernatant of *B. pseudomallei* 1710a, the proportion of flagellated *B. thailandensis* E264 cells was 43% (43/100), compared with 77%, 81% and 95% of *B. thailandensis* E264 cells exposed to supernatant of *B. pseudomallei* K96243, *B. thailandensis* E264 and non-exposed control, respectively (Figure 6). The flagella of *B. thailandensis* E264 cells exposed to *B. pseudomallei* 1710a supernatant appeared to be truncated, damaged and fragmented (Figure 6). In contrast, intact flagella were observed for *B. thailandensis* E264 cells exposed to supernatant of *B. pseudomallei* K96243 and the non-exposed control.

RT-PCR experiments was performed to measure the expression of the *fliC* gene in *B. thailandensis* E264 exposed to cell-free supernatant of *B. pseudomallei* 1710a, *B. pseudomallei* K96243, *B. thailandensis* E264 and non-exposed control. No difference in gene expression was observed (Figure 5F). These results suggest that the inhibition of *B. thailandensis* E264 motility is not due to reduction of *fliC* transcription but appears to be caused by flagellar processing or damage*.*

## Discussion

The observation that *B. pseudomallei* and *B. thailandensis* are rarely isolated from the same soil samples led us to examine whether these bacterial species compete in a range of in vitro model systems. We found that *B. pseudomallei* isolates inhibit growth of *B. thailandensis*. We demonstrated that clinical and environmental *B. pseudomallei* isolates with at least four different CDI types [10] suppressed *B. thailandensis* growth on LB agar plates. Notably, our results also demonstrated that 55% of *B. pseudomallei* tested secreted a protein, “inhibitory factor” that hindered *B. thailandensis* motility through flagellar processing such as assembly or secretion, or flagellar degradation. The inhibitory factor of *B. pseudomallei* is a heat-labile protein which functioned over a limited pH range. Our attempts to purify and identify the inhibitory factor from four liters of *B. pseudomallei* culture using gel-filtration and ion-exchange chromatography failed, which may have been due to low abundance and/or instability of the factor. Further studies using alternative approaches such as identification of the gene(s) coding for inhibitory factor and overexpression of the factor will be necessary for further characterization.

Studies in Lao PDR and Australia have revealed that soil collected in different seasons contain different numbers of *B. pseudomallei* [14,15]. Environmental dissemination of *B. pseudomallei* during the rainy season may involve motility [12,16]. Flagella are known to be the major cell-associated factor for bacterial motility, and *B. pseudomallei* mutants defective in the structural flagellin protein (FliC), do not swarm on agar [17]. Here, we found that swarming motility of *B. thailandensis* was inhibited by half of our *B. pseudomallei* isolates. Our microscopy results and RT-PCR data suggested that the inhibitory secreted product did not down-regulate flagellar expression, but led to structural changes. The mechanism is unknown but could involve several potential processes such as assembly, secretion, proteolytic degradation and depolymerization.

Analysis of genotype showed that the flagellar inhibitory factor was present in all of the isolates tested from 14 STs, and was absent in all isolates from 10 other STs, although 5 STs were present in both groups. Our test isolates included the two most abundant STs in Thailand (ST70 and ST54), which reside in different clonal complexes [18], and exhibited different inhibition results. Thus, the ability to secret the inhibitory compound was related to *B. pseudomallei* genotype. In contrast, we did not find a difference in inhibitory effect on *B. thailandensis* swarming motility for three isogenic morphotypes from each of five *B. pseudomallei* isolates, even though a previous study demonstrated that colony morphology variation represents several phenotypic differences [12].

One explanation for *B. pseudomallei* population structuring within a single soil sample is that the predominant clone may have a greater ability to inhibit *B. thailandensis* and minor populations of *B. pseudomallei*. We demonstrated that all *B. pseudomallei* isolates with the highest genotype frequency for each of three soil samples consistently inhibited motility of *B. thailandensis.* However, some minor populations also had this ability. The finding that the culture supernatant of all *B. pseudomallei* did not inhibit motility of any other *B. pseudomallei* suggests that the role of this secreted factor may be to inhibit the motility of other species. Flagellar filament of bacteria is composed of a flagellin subunit, which is diverse among bacterial species [19]. Bioinformatic analysis has demonstrated that the amino acid sequence of flagellin is conserved within each of species of *B. pseudomallei* and *B. thailandensis*. However, sequence alignment between several isolates of the two species has revealed a consistent 5 amino acid deletion at positions 247–251 (SPSFQ) in the flagellin of *B. thailandensis* [20]. In addition, there are 30 differences in amino acid distributed across the flagellin sequence. A recent report of the comparison between flagellin proteins of these two species also showed that their flagellins was modified with different masses of glycan (291 Da for *B. pseudomallei* and 300 or 342 Da for *B. thailandensis*) [21]. It is possible that these factors may be implicated in the different susceptibility to the inhibition activity of *B. pseudomallei*.

The ability of *B. pseudomallei* to survive in diverse environments is likely to be associated with a genetic repertoire that facilitates competition and adaptation [22]. This includes the presence of at least 10 CDI types that have been identified in the genomes of sequenced *B. pseudomallei* strains [10]. Our results are consistent with the hypothesis that the growth inhibition we observed on LB agar was caused by one or more CDI or T6SS systems, since *B. pseudomallei* culture supernatants had no effect on growth of *B. thailandensis*. Previous work demonstrated that *B. pseudomallei* CdiA-CT toxins are functional [10]. For example, the CdiA-CT of *B. pseudomallei* exhibits tRNase activity, which blocks the growth of *B. thailandensis* E264 target cells [10]. It is also possible that another mechanism of contact-dependent growth inhibition may be involved such as Type VI secretion systems (T6SS) that are present in *B. pseudomallei* [9].

Our analysis suggests that this growth inhibition phenomenon was not the result of different growth rates of *B. pseudomallei* and *B. thailandensis.* Inhibition of *B. thailandensis* by *B. pseudomallei* 1710a was only observed at a ratio greater than 1:1, suggesting that the *B. pseudomallei* inhibitor may mediate growth inhibition in a cell-density dependent or in a co-operative manner. Such cooperation has not been reported for *B. pseudomallei,* but it has been reported that the CDI genes in *B. thailandensis* were expressed in a subpopulation during culture in broth and also played a role in biofilm formation [11,23].

## Conclusion

Our results indicate that *B. pseudomallei* inhibits both the growth and motility of its close relative *B. thailandensis*. The latter phenomenon appears to occur via a previously unreported mechanism involving an effect on flagellar structure.

## Methods

### Bacterial isolates

Sixty-seven *B. pseudomallei* and six *B. thailandensis* isolates were examined in this study. The *B. pseudomallei* isolates originated from Thailand (thirty-six from human infection and twenty-seven from the environment) or Australia (three from human infection, and one from the environmental water) [4,24] (Additional file 1: Table S1). Five *B. thailandensis* (E29, E175, E264, E421 and E426) were isolated from soil in central Thailand and the sixth isolate was *B. thailandensis* Bt6, a kanamycin resistant mutant derived previously from *B. thailandensis* E264 (E264 *attTN7*::miniTn7T-Kan) [10]. A further fifteen *B. pseudomallei* strains were tested, which were derived from five isolates (153, 164 & K96243 from human disease in Thailand, B3 and B4 from the environment in Thailand) [4,12] (Additional file 1: Table S1). This included wild type (type I), together with two isogenic colony morphology variants (types II & III) for each isolate, which were generated from type I of each strain using nutritional limitation [12,13]. Bacteria were grown in Luria-Bertani (LB) broth or on LB agar and incubated at 37°C in air. LB with 500 μg/ml kanamycin (Kan) was used to culture *B. thailandensis* Bt6. The study was granted exemption from requiring ethics approval by Ethics Committee of Faculty of Tropical Medicine, Mahidol University.

### Growth curves

A single bacterial colony was suspended in sterile phosphate buffered saline (PBS) and adjusted to an OD_600_ of 0.15 to obtain 1 × 10^8^ cfu/ml. One hundred microlitres of bacterial suspension was added to 10 ml of LB broth and incubated at 37°C in air with shaking at 200 rpm for 24 h. At 2 h intervals, 100 μl of bacterial culture was collected, serially diluted 10-fold in PBS, and the number of viable cells counted by plating on LB agar or LB agar containing 500 μg/ml Kan (for *B. thailandensis* Bt6) in triplicate. Plates were incubated at 37°C in air for 2 days and doubling time was calculated.

### Growth inhibition determined by broth microdilution assay

A broth microdilution assay was used to determine the effect of cell-free supernatant from *B. pseudomallei* on growth of *B. thailandensis. B. pseudomallei* supernatant from overnight culture in LB broth at 37°C was concentrated using a filter membrane with 10,000 cut-off (Millipore, Billerica, MA, USA) to 5×, 2.5× and 1.2×, and an equal volume of each concentrate added to 50 μl of an overnight culture of *B. thailandensis* to obtain a final concentration 5 × 10^5^ cfu/ml in LB. The control was performed by adding an equal volume of PBS to 50 μl of an overnight culture of *B. thailandensis.* The experiment was performed in a 96-well plate in triplicate in a given experiment. Cultures were incubated at 37°C in air for 18 h. Bacterial growth was defined as visual turbidity followed by colony count.

### Swarming motility inhibition assay

Three methods were devised to test whether interactions between *B. pseudomallei* and *B. thailandensis* affected swarming motility. (i) A colony of *B. pseudomallei* and a colony of *B. thailandensis* obtained from an overnight culture on LB agar were spotted using a pipette tip at opposite poles of a swarm agar plate, and the growth observed daily over a 3 day incubation time course at 37°C. (ii) Supernatant from an 18 hour *B. pseudomallei* culture in LB broth was harvesting using centrifugation and filtrated through 0.2 μm membrane (Sartorius, Goettingen, Germany). A colony of *B. thailandensis* from overnight culture on LB agar was spotted onto the center of the plate and incubated at 37°C for 16 h, after which 100 μl of cell-free *B. pseudomallei* supernatant was dropped onto the plate at 2, 6 and 10 o’clock and 1–1.5 cm from the edge of the plate. The plate was further incubated for 16 h, and then observed for inhibition of *B. thailandensis* swarming, which was defined as the presence of any clear zone around the supernatant drop. We spotted the supernatant of *B. pseudomallei* after *B. thailandensis* was allowed to swarm for a period of time because the inhibition activity was lost if applied at the beginning of the assay. (iii) 100 μl of cell-free *B. pseudomallei* supernatant was dropped in the center of a swarm plate and left to diffuse and dry in air for 15 min. The same spot was then inoculated with a *B. thailandensis* colony using a pipette tip, and the plate then incubated for 18 h before the swarm *B. thailandensis* colony was examined.

### Growth inhibition assay on agar plates

A growth inhibition assay was carried out as described previously [10]. In brief, bacteria were grown for 4 h to early log phase (OD 600 nm = 0.2-0.5) in LB or LB with Kan. Bacterial concentration was adjusted using OD, *B. pseudomallei* mixed with *B. thailandensis* in a ratio of 1000:1, 100:1, 10:1 or 1:1, and 100 μl of the mixture dropped onto a 2.5 × 2.5 cm^2^ sterile nitrocellulose membrane which was placed on top of LB agar. Plates were incubated at 37°C in air for 24 h after which the nitrocellulose membrane was removed, placed into 10 ml PBS and vortexed vigorously for 30 sec to harvest bacteria. Duplicate plating onto LB and LB plus Kan was performed, and plate counts determined for *B. thailandensis* Bt6 (LB/Kan plate) and *B. pseudomallei* (total count on LB minus *B. thailandensis* count on LB/Kan). To examine the effect of incubation time, a co-culture of *B. pseudomallei* 1710a and *B. thailandensis* Bt6 at a ratio of 1000:1 was incubated at 37°C for 3 h, 6 h and 24 h. At the indicated time point, cells were harvested from the plates and viable bacteria counted as before. The interaction was also tested between *B. thailandensis* Bt6 and six *B. pseudomallei* isolates (K96243, H1244a, 1106b, 1026b, 1710a, B4), in which *B. thailandensis* E264 wild type was used for comparison using a ratio of *B. thailandensis* E264 to *B. thailandensis* Bt6 at 1000:1 for 24 h. Three *B. pseudomallei* isolates possessed different CDI types (strains K96243, type I; 1710a, type II; and 1026b, type V and VIII) [10]. All assays were performed in triplicate in a given experiment.

### Separation and preparation of concentrated motility inhibitory factor

*B. pseudomallei* was inoculated into LB and incubated overnight at 37°C in air with shaking at 200 rpm. The bacterial culture was centrifuged at 4,000 × g for 15 min at 4°C and the supernatant filtrated through a 0.2 μm membrane. To estimate the molecular size of the inhibitory substance, 10 ml of the cell-free supernatant of *B. pseudomallei* 1710a was filtrated through filter membranes with a molecular weight cut-off (MWCO) of 10,000, 30,000, 50,000 or 100,000 daltons (Millipore, Billerica, MA, USA) to obtain 15–100 times concentrated culture supernatant. The presence of inhibitory substance in the retentate or filtrate after membrane separation was assessed using the inhibition motility assay (method (ii)).

### Protein digestion of inhibitory molecule

The protein nature of the inhibitory factor was characterized by digestion with two different proteases. Cell-free supernatant of 18 h culture at 37°C in LB broth of *B. pseudomallei* 1710a was concentrated 15 times using 30,000 membrane filtration, quantified using the bicinchoninic acid assay (Pierce, Rockford, IL, USA), and analyzed by SDS-PAGE [25]. The sample was diluted to 1 mg/ml and digested at an enzyme:substrate ratio of 1:10 (w/w) with proteinase K (Invitrogen, Carlsbad, CA, USA) or pronase (Merck, Nottingham, UK) in the relevant buffer as recommended by the manufacturer (proteinase K in PBS at 37°C for 72 h and pronase in PBS containing 10 mM CaCl_2_ at 37°C for 72 h).

### Assays for stability of inhibitory factor

Inhibition activity of cell-free supernatant of *B. pseudomallei* 1710a was tested after adjustment to a pH of 3, 4, 5, 6, 7 or 8, and after being maintaining at pH 7.0 at –80°C, 4°C, 25°C, 37°C, 50°C or 80°C for 24 h. Inhibition activity was also tested at pH 7.0 in the presence of NaCl at a final concentration of 0.2 M, 0.4 M, 0.6 M, 0.8 M or 1 M. Aliquots of 100 μl were tested in the *B. thailandensis* E264 inhibition motility assay at 37°C for 18 h (method ii). Untreated overnight cell-free supernatant of *B. pseudomallei* 1710a in LB broth was used as a positive control and LB broth was used as a negative control. Results for inhibition of *B. thailandensis* motility were read based on the presence (positive) or absence (negative) of a clear zone on swarm agar.

### Visualization of *B. thailandensis* motility and flagella

Live cell imaging was used to examine the motility of *B. thailandensis* E264 after exposure to cell-free supernatant from *B. pseudomallei* 1710a, *B. pseudomallei* K96243 and *B. thailandensis* E264 (control). The method was modified from a previous study [26]. *B. thailandensis* cells were exposed to *B. pseudomallei* cell-free supernatant using the swarming inhibition motility method (iii). *B. thailandensis* E264 was picked from the colony at the end of this assay and suspended in 50 μl PBS. Two microlitres of bacterial suspension was overlaid on a film of 1.5% low-melting agarose pad (Invitrogen, Carlsbad, CA, USA) on a microscope glass slide. A cover slip was placed and motility was observed by light microscope (Leica DM750, Wetzlar, Germany) with 100× magnification. Video was recorded and 20 individual cells were tracked and analyzed for 20 sec motility using ImageJ program (http://rsb.info.nih.gov/ij/) with the manual tracking plug-in.

The same *B. thailandensis* E264 cell preparation was examined using TEM for the presence of flagella. Fifty microliters of the *B. thailandensis* suspension was dropped onto parafilm and Fomvar coated carbon grids were placed on top for 10 min to transfer bacterial cells. The liquid was carefully removed with filter paper and the samples were stained with 1% uranyl acetate for 10 min after which the liquid was again carefully removed. The grid was dried at room temperature for overnight. Bacteria were observed with a Hitachi Electron Microscope H-7000 (Japan). The presence of bacterial flagella was recorded for 100 bacteria per isolate.

### Reverse transcriptase PCR for *fliC* gene expression

*B. thailandensis* E264 cells were picked from a colony at the completion of method (iii) as above. RNA was extracted using Trizol reagent, as described previously [27]. Primers were designed for *fliC* of *B. thailandensis* E264 (accession number AF081500.1) using Primer-BLAST (http://www.ncbi.nlm.nih.gov/tools/primer-blast). One-step reverse transcriptase (RT)-PCR was performed using 1 μg RNA and a Superscript III One-step RT-PCR system (Invitrogen, Carlsbad, CA, USA) with forward primer 5′ GGTCGCTCAACAGAACCTCA 3′ and reverse primer 5′ CTGGTTCAGGCCGTTGATCT3′. The RT-PCR conditions were as follows: cDNA synthesis at 45°C for 30 min; initial denaturation at 95°C for 5 min; 30 cycles of denaturation at 94°C for 30 sec, annealing at 50°C for 15 sec, and extension at 72°C for 30 sec; and a final elongation step at 72°C for 7 min. The positive control was RT-PCR for 23S rRNA amplification using primers 23S_F and 23S_R with forward primer 5′ GTAGACCCGAAACCAGGTGA3′ and reverse primer 5′ CACCCCTATCCACAGCTCAT 3′. The negative control was a reaction without RT enzyme. The amplified product was run on a 1.5% agarose gel, stained with ethidium bromide and visualized under UV light.

### Statistical analysis

Statistical analyses were performed using Stata, version 12 (StataCorp LP, College Station, TX, USA). Fisher’s exact test or Chi square tests were used to test proportions. One-way ANOVA was used to test the difference between groups. Quantitative data are presented as mean ± standard deviation. Differences were considered statistically significant at a p-value ≤ 0.05.

## Additional file

Additional file 1: Table S1. Summary of *B. pseudomallei* isolates and inhibitory activity against *B. thailandensis* swarming motility.
