# Supplementary material for: Competition between Burkholderia pseudomallei and B. thailandensis
Source: BMC Microbiol. 2015 Mar 3;15:56. doi: 10.1186/s12866-015-0395-7 (PMC4365494; doi:10.1186/s12866-015-0395-7)
Supplement: Additional file 1: Table S1. — Summary of B. pseudomallei isolates and inhibitory activity against B. thailandensis swarming motility. [file 12866_2015_395_MOESM1_ESM.docx]

**Table S1** Summary of *B. pseudomallei* isolates and inhibitory activity against *B. thailandensis* swarming motility

| **No.** | **Bp isolates** | **Sequence**  **type** | **Country of origin** | **Source** | **Specimen** | **Inhibition effect*** |
| --- | --- | --- | --- | --- | --- | --- |
| 1 | K 96243 | 10 | Thailand | Clinical | Blood | - |
| 2 | 153 | 15 | Thailand | Clinical | Blood | + |
| 3 | B1 | 33 | Thailand | Environment | Soil (unused land) | + |
| 4 | E411 | 54 | Thailand | Environment | Soil (rice field) | + |
| 5 | H1882a | 54 | Thailand | Clinical | Pus | + |
| 6 | H2613a | 54 | Thailand | Clinical | Blood | + |
| 7 | H2659a | 54 | Thailand | Clinical | Respiratory secretion | + |
| 8 | H2660a | 54 | Thailand | Clinical | Blood | + |
| 9 | H2670a | 54 | Thailand | Clinical | Respiratory secretion | + |
| 10 | SBPTHE0411 | 54 | Thailand | Environment | Soil (rice field) | + |
| 11 | SBPTHE0021 | 54 | Thailand | Environment | Soil (rice field) | + |
| 12 | SBPTHE0235 | 54 | Thailand | Environment | Soil (rice field) | - |
| 13 | SBPTHE0413 | 54 | Thailand | Environment | Soil (rice field) | + |
| 14 | SBPTHE0024 | 54 | Thailand | Environment | Soil (rice field) | + |
| 15 | SBPTHE0037 | 54 | Thailand | Environment | Soil (rice field) | + |
| 16 | SBPTHE0399 | 54 | Thailand | Environment | Soil (unused land) | + |
| 17 | B2 | 60 | Thailand | Environment | Soil (unused land) | + |
| 18 | C4 | 60 | Thailand | Environment | Soil (unused land) | + |
| 19 | H1244a | 60 | Thailand | Clinical | Pus | + |
| 20 | H1248a | 60 | Thailand | Clinical | Pus | + |
| 21 | H2708a | 60 | Thailand | Clinical | Pus | + |
| 22 | H2820a | 60 | Thailand | Clinical | Pus | - |
| 23 | SBPTHE0378 | 60 | Thailand | Environment | Soil (unused land) | - |
| 24 | SBPTHE0383 | 60 | Thailand | Environment | Soil (unused land) | - |
| 25 | SBPTHE0031 | 60 | Thailand | Environment | Soil (rice field) | + |
| 26 | 1106a | 70 | Thailand | Clinical | Pus | - |
| 27 | 1106b | 70 | Thailand | Clinical | Pus | - |
| 28 | E371 | 70 | Thailand | Environment | Soil (unused land) | - |
| 29 | H2614a | 70 | Thailand | Clinical | Body fluid | + |
| 30 | H2635a | 70 | Thailand | Clinical | Blood | - |
| 31 | H2663a | 70 | Thailand | Clinical | Wound swab | - |
| 32 | H2664b | 70 | Thailand | Clinical | Blood | - |
| 33 | H2677a | 70 | Thailand | Clinical | Respiratory secretion | - |
| 34 | H2684a | 70 | Thailand | Clinical | Pus | - |
| 35 | H2689b | 70 | Thailand | Clinical | Blood | - |
| 36 | H2693a | 70 | Thailand | Clinical | Respiratory secretion | - |
| 37 | H2694a | 70 | Thailand | Clinical | Respiratory secretion | - |
| 38 | H2747a | 70 | Thailand | Clinical | Blood | - |
| 39 | H1794a | 70 | Thailand | Clinical | Pus | - |
| 40 | H1814a | 70 | Thailand | Clinical | Pus | + |
| 41 | H2454a | 70 | Thailand | Clinical | Pus | - |
| 42 | H2644a | 70 | Thailand | Clinical | Blood | - |
| 43 | SBPTHE0038 | 70 | Thailand | Environment | Soil (rice field) | - |
| 44 | SBPTHE0358 | 70 | Thailand | Environment | Soil (rice field) | - |
| 45 | SBPTHE0359 | 70 | Thailand | Environment | Soil (rice field) | - |
| 46 | C1 | 93 | Thailand | Environment | Soil (unused land) | + |
| 47 | 1026b | 102 | Thailand | Clinical | Blood | + |
| 48 | MSHR491 | 126 | Australia | Environment | Water | - |
| 49 | MSHR435 | 126 | Australia | Clinical | No data | - |
| 50 | MSHR668 | 129 | Australia | Clinical | Blood | - |
| 51 | MSHR465a | 132 | Australia | Clinical | No data | + |
| 52 | B3 | 163 | Thailand | Environment | Soil (unused land) | + |
| 53 | A3 | 176 | Thailand | Environment | Soil (unused land) | + |
| 54 | B4 | 176 | Thailand | Environment | Soil (unused land) | - |
| 55 | 1710a | 177 | Thailand | Clinical | Blood | + |
| 56 | 1710b | 177 | Thailand | Clinical | Blood | + |
| 57 | E645 | 177 | Thailand | Environment | Soil (unused land) | + |
| 58 | A2 | 177 | Thailand | Environment | Soil (unused land) | + |
| 59 | H1799a | 177 | Thailand | Clinical | Wound swab | + |
| 60 | H1807a | 177 | Thailand | Clinical | Pus | - |
| 61 | H2676a | 177 | Thailand | Clinical | Wound swab | + |
| 62 | A4 | 185 | Thailand | Environment | Soil (unused land) | + |
| 63 | 406e | 211 | Thailand | Clinical | Wound swab | + |
| 64 | C2 | 304 | Thailand | Environment | Soil (unused land) | - |
| 65 | A1 | 424 | Thailand | Environment | Soil (unused land) | - |
| 66 | 576a | 501 | Thailand | Clinical | Blood | + |
| 67 | 164 | No data | Thailand | Clinical | Blood | + |

***** Inhibition effect of *B. pseudomallei* cell-free supernatant on *B. thailandensis* isolates. The inhibition of *B. thailandensis* swarming was performed by all three types of assays and the same results were obtained. The table shows results of five *B. thailandensis* isolates (E29, E175, E264, E421 and E426) which had the same results.
